# Highly specific neutrophil-mediated delivery of albumin nanoparticles to ectopic lesion for endometriosis therapy

**DOI:** 10.1186/s12951-023-01831-4

**Published:** 2023-03-08

**Authors:** Shasha Zhu, Jiqian Zhang, Nairui Xue, Xiaoling Zhu, Fenfen Li, Qingqing Dai, Xin Qing, Dawei Chen, Xuesheng Liu, Zhaolian Wei, Yunxia Cao

**Affiliations:** 1 Reproductive Medicine Center, Department of Obstetrics and Gynecology, The First Affiliated Hospital of Anhui Medical University, Hefei, 230032 China; 2 NHC Key Laboratory of Study On Abnormal Gametes and Reproductive Tract, No 81 Meishan Road, Hefei, 230032 Anhui China; 3 Key Laboratory of Population Health Across Life Cycle (Anhui Medical University), Ministry of Education of the People’s Republic of China, No 81 Meishan Road, Hefei, 230032 Anhui China; 4 Department of Anesthesiology, The First Affiliated Hospital of Anhui Medical University, Key Laboratory of Anesthesiology and Perioperative Medicine of Anhui Higher Education Institutes, Anhui Medical University, Hefei, 230032, Anhui China; 5 Hefei National Lab for Physical Sciences at the Microscale and Centers for Biomedical Engineering, University of Science and Technology of China, Hefei, 230032, Anhui China

**Keywords:** Endometriosis, Neutrophils, Bovine serum albumin nanoparticles, Glucose oxidase

## Abstract

Endometriosis is an estrogen-dependent chronic inflammatory disease. Hormonal and surgical treatments are the most commonly used clinical therapies, but they have many sides effects or are traumatic to the body. Therefore, specific drugs for endometriosis treatment are urgently needed to develop. In this study, we identified two features of endometriosis, namely the continuous recruitment of neutrophils into the ectopic lesions and the higher uptake of glucose by ectopic cells. For the above features, we designed a glucose oxidase-loaded bovine serum albumin nanoparticle (BSA-GOx-NPs) that is inexpensive and facilitates large-scale production. After injection, BSA-GOx-NPs were high specifically delivered to ectopic lesions in a neutrophil-dependent manner. Furthermore, BSA-GOx-NPs deplete glucose and induce apoptosis in the ectopic lesions. Whereupon BSA-GOx-NPs produced excellent anti-endometriosis effects when administrated in both acute and chronic inflammatory phases. These results reveal for the first time that the neutrophil hitchhiking strategy is effective in chronic inflammatory disease and provide a non-hormonal and easy-to-achieve approach for endometriosis treatment.

**Supplementary Information:**

The online version contains supplementary material available at 10.1186/s12951-023-01831-4.

## Introduction

Endometriosis is an estrogen-dependent chronic inflammatory disease characterized by the presence of endometrial-like tissue outside the uterus. Endometriosis affects nearly 10% of women in their reproductive years, translating to billions of affected women worldwide [1, 2]. Pelvic pain and infertility are two major clinical symptoms of endometriosis that profoundly affect the quality of life of affected women [3]. To date, the etiology and pathogenesis of endometriosis remain elusive. A variety of factors including genetics, environment, immunity, endocrinology, and microorganisms are reported to be associated with the development of endometriosis [1]. However, there is no consensus on whether these factors influence endometriosis pathophysiology individually or in combination. Currently, surgical and hormonal treatments are still the most commonly used clinical therapies for women with endometriosis. Unfortunately, the estimated recurrence of endometriosis after surgical or hormonal treatment is up to 21.5% at 2 years and 40–50% at 5 years [4]. Furthermore, these treatments are accompanied by undesirable side effects including mood swings, pseudomenopause, bone density loss, and a higher risk of osteoporosis. Therefore, the development of new medical options with effective suppression of endometriosis and minimal adverse effects is desperately needed for the benefit of women suffering from this disease.

The immune system plays a central role in the etiology and pathophysiology of endometriosis [5–7]. As an important component of the innate immune system, neutrophils have attracted increasing attention in endometriosis research. It has been proved that neutrophil infiltration was increased in the systemic circulation and peritoneal fluid of endometriosis patients compared to that in disease-free women [8–11]. In addition, systemic circulating neutrophils from endometriosis patients possess a unique transcriptomic profile compared to neutrophils in healthy controls [9]. This evidence suggests neutrophils may play a critical role in the treatment of endometriosis. However, two groups have tested the effect of antibody-mediated neutrophil depletion in endometriosis, but the results are controversial [8, 9]. In view of the above facts, whether neutrophils are suitable as a therapeutic target and how to refine neutrophil-based therapeutic approach to make them more effective still need to be addressed.

Nanotechnology offers the possibility of treating clinically insurmountable diseases. For instance, Yang et al. developed the iodine (I)-polyvinyl alcohol (PVA)@polydopamine (PDA) microspheres to achieve the computed tomography image, drug loading and controlled release, and the enhanced embolization of liver portal vein [12]. Zhang et al. designed a polydopamine-modified carboxymethyl cellulose hydrogel for anti-recurrence of tumor. This hydrogel is biocompatible and biodegradable, has a good photothermal conversion, drug loading and CT imaging ability, which laid the foundation for the rational design of biodegradable hydrogels for multifunctional applications [13]. Neutrophil-based nanoplatforms are an effective strategy for the treatment of various diseases [14–18]. Specifically, neutrophil-membrane-derived nanovesicles are one of the most appealing neutrophil-based nanoplatforms. Generally, neutrophil-membrane-derived nanovesicles are prepared by fusing neutrophil membranes onto synthetic cores. Taking advantage of the antigenic profile of the source neutrophils, neutrophil-membrane-derived nanovesicles can act as cytokine sponges that neutralize proinflammatory cytokines [19–21]. Previous studies demonstrate that neutrophil-membrane-derived nanovesicles exert significant anti-tumor effects and alleviate many inflammatory diseases such as rheumatoid arthritis and sepsis, regardless of whether they are loaded with therapeutic drugs [19–23]. Another promising neutrophil-based nanoplatform is the application of hitchhiking neutrophils. For hitchhiking neutrophils, nanodrugs are usually internalized into neutrophils mediated by the molecules on neutrophils surface, and then they are delivered to the target tissues. Chu et al. reported anti-CD11b antibody-decorated NPs (NPs-CD11b) hitchhike neutrophils in vivo though CD11b-mediated internalization and then are delivered into the tumor tissues after acute inflammation induced by photosensitization [24]. Li et al. reported pathogen-mimetic nano-pathogenoids (NPNs) that can hitchhike neutrophils in situ by effectively recognized and internalized by neutrophils via toll-like receptors (TLRs) and subsequently be delivered to PTT-induced acute inflamed tumors [25]. What’s more, BSA nanoparticles were reported to hitchhike neutrophils and were delivered to the sites of acute inflammation, such as inflamed endothelium or lung [16, 17]. Furthermore, the internalization of BSA nanoparticles into neutrophils was attributed to the Fcγ receptors on the surface of neutrophils [16]. Although the above researches have reported that the nanodrugs can hitchhike neutrophils to the sites of acute inflammation, whether the hitchhiking neutrophil platform could apply to deliver nanodrugs to a chronic inflammatory site, such as ectopic lesions in endometriosis, remains to be tested.

At present, only a few reports describe the treatment of endometriosis with the help of nanotechnology [26–30]. Guo et al. designed a TNYL peptide conjugated hollow gold nanosphere (TNYL-HAuNS) that exhibited superior PTT efficacy under near-infrared (NIR) laser mediation [26]. In addition, activatable silicon naphthalocyanine (SiNc)-encapsulated polyethylene glycol-polycarpolactone (PEG-PCL) nanoparticles constructed by Moses et al. were able to efficiently delineate endometriotic lesions with NIR fluorescence signals and rapidly eliminate them by PTT [27]. Despite these advances, the limited tissue penetration of nanoparticle-based photothermal therapies should be noticed. The intensity of light required for the activation of currently available photosensitizers can only be achieved intraoperatively, meaning the use of nanoparticle-based PTT for the treatment of endometriosis may be limited to use as a component of surgical intervention [31]. Besides nanoparticle-based photothermal therapies, nanoparticle-based gene therapies, including chitosan derivatives micelle encapsulating pigment epithelium derived factor (PEDF) plasmid, and hyaluronic acid (HA) modified polyethylenimine-grafted chitosan oligosaccharide (CSO-PEI) compressing siRNA system, have also been shown to effectively inhibit endometriosis [29, 30]. However, limitations in the safety and suitability for reproducible large-scale manufacturing hinder the clinical translation of these nanoparticle-based therapies.

**Fig. 1:**
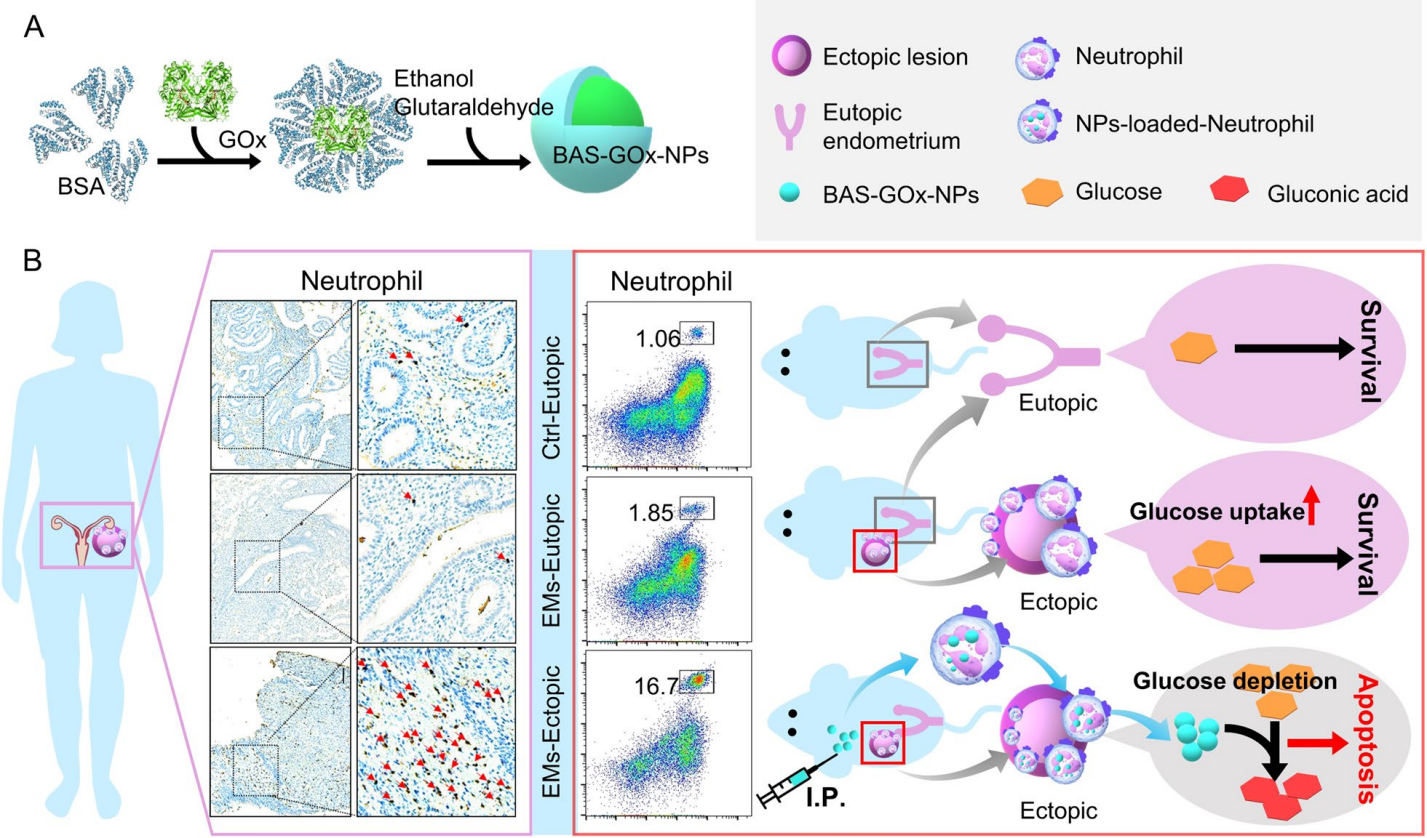
Schematic Illustration of the Anti-Endometriosis Effects of BSA-GOx-NPs. **A** Scheme showing the synthesis of BSA-GOx-NPs by desolventization. **B** Neutrophils were enriched in both human and mouse endometriosis lesions. BSA-GOx-NPs flowing intraperitoneal injection hitchhike neutrophils and be delivered to ectopic lesions. Ectopic stromal cells have a higher demand for glucose than eutopic stromal cells. Following intraperitoneal injection of BSA-GOX-NPs, glucose oxidase is released and consumes glucose in ectopic lesions, ultimately relieving endometriosis

Given the current dilemma, we hypothesized that developing a safe and effective nanomedicine to hitchhike neutrophils may be a promising strategy to target ectopic lesions and thus inhibit endometriosis. Therefore, we first examined the changes of neutrophils in human and mice ectopic lesions and conclusively demonstrated a sustained enrichment of neutrophils at the lesion. In addition, albumin NPs are considered to be biodegradable, non-toxic, non-immunogenic, easy-to-prepare and reproducible nanomedicines, and they have been approved by FDA for clinical use [32–35]. Moreover, albumin NPs synthesized with organic solvents (such as ethanol) are reported to specifically bind to activated neutrophils and thus be transported to acutely inflamed tissues [16, 17], but the delivery of albumin NPs to chronically inflamed tissues by neutrophils have not been reported. Hence, we synthesized homogeneous BSA-NPs with a size of approximately 300 nm using a desolvation method. Then, in a mouse model of endometriosis (a chronic inflammatory disease), we demonstrated that intraperitoneally injected BSA-NPs were internalized by neutrophils in vivo and highly specifical enriched in ectopic lesions in a neutrophil-dependent manner. Furthermore, a higher demand for glucose was detected in ectopic stromal cells compared to eutopic stromal cells. Therefore, we synthesized BSA-GOx-NPs by loading glucose oxidase (GOx) into BSA-NPs and verified that the enzymatic activity and cytotoxicity of GOx were preserved in the BSA-NPs. Whereafter, we found that intraperitoneal injection of BSA-GOx-NPs produced an excellent anti-endometriosis effect by inducing eutopic lesions apoptosis with undetectable side effects (Fig. 1).

## Materials and methods

### Materials

Estradiol benzoate (HY-B1192) was purchased from MCE. Gox (G7141), collagenase type IV (C5138) and DNase I (11284932001) were purchased from Sigma-Aldrich. Fluorochrome-labeled or unlabeled monoclonal antibodies against mouse Ly6G (108454, 127607), IgG2b isotype control (400675), CD16/32 (101319), CD45 (103113), CD11b (101227), F4/80 (123115), FcγR III (158013) were purchased from BioLegend. Recombinant anti-myeloperoxidase antibody (ab208670) was purchased from Abcam. DMEM/F-12 GlutaMAX (SH30023.01) was purchased from Hyclone. 2-NBDG (N13195) was purchased from Invitrogen. Cell Counting Kit-8 (E-CK-A362) was purchased from Elabscience. BSA-FITC, BSA-ICG were purchased from Xi’an ruixi Biological Technology Co., Ltd.

### Mice

Six to eight weeks old female Balb/c mice, weighing 18–25 g, were purchased from the GemPharmatech Co., Ltd. All animals were housed in temperature, humidity, and light-controlled rooms, with water provided ad libitum. Animal welfare and experimental procedures were carried out in accordance with the Ethical Regulations on the Care and Use of Laboratory Animals of Anhui Medical University and were approved by the school committee for animal experiments (LLSC20200083).

### Clinical biospecimens

Clinical ectopic endometriosis biospecimens were obtained from six endometriosis patients from six patients with ovarian endometriosis. Matched endometrial samples (eutopic) were collected from the same endometriosis patients. Similarly, endometrial biopsies were collected from six healthy, fertile controls at the first affiliated hospital of Anhui Medical University. Ethical approval was obtained from the Ethics Committee of Anhui Medical University. Informed consent was obtained.

### Model of endometriosis

The mouse model of endometriosis was performed as described previously [36, 37]. Donor mice were treated subcutaneously with estradiol benzoate (3 μg/mouse, MCE). One week after the estrogen injection, donor mice were sacrificed and each uterine horn was collected and split longitudinally with a pair of scissors. Carefully mechanical dissected each uterine horn into fragments with a maximal diameter lower than 1 mm, Endometriosis was induced by injecting the uterine horn fragments from two mice intraperitoneally into three recipient mice.

### Synthesis and characterization of BSA-NPs, BSA-GOx-NPs, FITC- BSA-NPs, ICG-BSA-NPs and FITC- BSA-GOx-NPs, ICG-BSA-GOx-NPs

BSA-GOx-NPs and BSA-NPs were synthesized by referring to and improving previously reported methods [17, 38]. Briefly, 1 mL BSA aqueous solution (10 mg/mL) was mixed with or without 100 μL GOx (G7141, Sigma-Aldrich) aqueous solution (10 mg/mL) for 1 h. Next, 3 mL ethanol was added dropwise into the above solution under 600 r.p.m. stirring for 1 h. Afterward, 10 μL 2.5% glutaraldehyde was added to the mixture, stirring for 6 h at room temperature. Then, the above solution was centrifuged at 14,000 r.p.m. for 20 min at 4 °C. The precipitate was centrifuged for three times to remove organic solvents and then was re-suspended in 1 mL Milli-Q water and ultrasound for further use. The morphology of BSA-GOx-NPs and BSA-NPs were characterized by transmission electron microscope (JEOL-2010). The particle size distribution and zeta potential of BSA-GOx-NPs and BSA-NPs were determined using dynamic light scattering (DLS) with a particle size analyzer (90 Plus, Brookhaven Instruments Co.). The synthesis of FITC- BSA-NPs, ICG-BSA-NPs or FITC- BSA-GOx-NPs, ICG-BSA-GOx-NPs was similar to the above-mentioned, except that 1 mL of 10 mg/ml BSA aqueous solution was changed to a mixture of 1 mL of 10 mg/ml BSA aqueous solution and 100 μL of 10 mg/ml FITC- BSA or ICG-BSA aqueous solution.

### Synthesis of FITC labeled GOx (GOx-FITC)

GOx (10 mg) was mixed with fluorescein isothiocyanate (FITC, 1 mg) in 100 mM NaHCO_3_ buffer (pH 8.5, 5 mL) for 24 h at 25 °C. Excessive FITC was removed by ultrafiltration centrifugation (Millipore, 50 kDa MWCO).

### Encapsulation efficiency and drug loading

To calculate theencapsulation efficiency (EE%) and drug loading (DL%) of GOx into BSA-NPs, 100 μL GOx -FITC aqueous solution (10 mg/mL) was mixed with 1 mL BSA aqueous solution (10 mg/mL) to prepare BSA-GOx-FITC-NPs. During the fabrication process of BSA-GOx-FITC-NPs, the supernatant was collected after centrifugation and the amount of unbound in comparison to originally added GOx-FITC (m_free GOx-FITC_ and m_total GOx-FITC_, respectively) was determined at an excitation and emission wavelength of 488 and 528 nm, respectively, using a fluorescence microplate reader (Varioskan Flash, Therm Scientific). Encapsulation efficiency (EE%) and drug loading (DL%) of GOx -FITC into BSA NPs were calculated according to the following equations:

EE% = (m_total GOx-FITC_—m_free GOx-FITC_) / m_total GOx-FITC_ × 100%

DL% = (m_total GOx-FITC_—m_free GOx-FITC_) / m_NPs_ × 100%

### Catalytic activity measurements of BSA-GOx-NPs

The production of H_2_O_2_ by BSA-GOx-NPs, glucose, and oxygen was determined via a classic colorimetric method, applying ammonium titanyl oxalate as the indicator. In brief, 0.1 mL of BSA-GOx-NPs or BSA-NPs suspension (50 μg/mL BSA) was mixed with 0.1 mL of different glucose concentrations (0, 0.025, 0.05, 0.1, 0.2, 0.5, 1 and 2 mg/mL). After 1 h, ammonium titanyl oxalate solution (10 μL, 10 mM) was added. The obtained yellow suspension was measured by a microplate reader (Nano Quant, Tecan) at 405 nm.

### Neutrophil depletion in mice

To deplete neutrophils, mice were treated with a 400 μg intraperitoneal injection of anti-mouse Ly6G antibody (108454, Biolegend) or IgG2b isotype control (400675, Biolegend) 24 h prior to injection of BSA-NPs. Subsequent 200 μg antibody injections were administered accompanied by BSA-NPs. Animals were euthanized after 16 h to collect peritoneal lavage fluid, ectopic lesions, and eutopic endometrium. Neutrophil depletion was confirmed by flow cytometry as described below.

### Eutopic stromal cell and ectopic stromal cell isolation and flow cytometric analyses

Procedures were principally performed as described [39]. Briefly, murine uteri were minced into small pieces and incubated in PBS containing 1 mg/mL collagenase type IV and 40ug/ml DNase I for 45 min at 37 °C with shaking at 100 rpm. For flow cytometric analyses, cell suspensions were incubated with anti-CD16/32 for Fc blocking (101319, BioLegend), and stained with PE/cyanine7-conjugated anti-CD45 (103113, BioLegend), PerCP/Cyanine5.5-conjugated anti-CD11b (101227, BioLegend), and PE-conjugated anti-Ly6G (127607, BioLegend) mAbs for 30 min on ice. For eutopic stromal cell and ectopic stromal cell isolation, cell suspensions were strained by using 70 μm nylon cell strainers. The generated cell suspensions were strained by using 70 μm and 40 μm nylon cell strainers and twice washed by centrifugation at 300 g. The final pellet containing the endometrial stromal cells was cultured at 37 °C with 5% CO_2_ in the DMEM/F-12 GlutaMAX (SH30023.01, Hyclone) medium supplemented with 10% fetal bovine serum (FBS) and the antibiotic/antimycotic mix. The cells were allowed to adhere for 24 h, and non-adherent cells were removed by replacing the medium. Upon reaching 70–90% confluence, adherent cells were harvested by trypsinization and subcultured at a 1:3 ratio. To measure glucose uptake, eutopic stromal cells and ectopic stromal cells were incubated with 100 mM 2-NBDG (N13195; Invitrogen) for 10 min at 37 ℃. Samples were acquired by a BD FACSVerse flow cytometry, and data were analyzed with FlowJo software (TreeStar).

### Cell viability assay

Ectopic stromal cells were seeded in 96-well plates (2 × 10^4^ cells/well) and cultured at 37 °C with 5% CO_2_. 24 h later, ectopic stromal cells were incubated with different concentrations of GOx and BSA-GOx-NPs for another 12 h. 10 μL Cell Counting Kit-8 (E-CK-A362, Elabscience) was added to each well and incubated at 37 °C for 30 min. Then, the absorbance of plate was measured at 450 nm using a microplate reader (Nano Quant, Tecan).

### In Vivo* therapy*

Mice with endometriosis were randomly divided into three groups: 1. Control treatment animals received an intraperitoneal injection of 100 μl sterile PBS; 2. BSA -NPs treatment mice were intraperitoneally injected with 95 μl sterile PBS + 5 μl 10 mg/mL BSA -NPs; 3. BSA-GOx-NPs treatment mice were intraperitoneally injected with 95 μl sterile PBS + 5 μl 10 mg/mL BSA-GOx-NPs. Treatment in the acute inflammatory phase of endometriosis started from day 3 post donor endometrial fragments injection, while treatment in the chronic inflammatory phase begun on day 14 after injecting the donor mouse endometrial fragments. Mice were treated every other day, 3 times/week for 2 weeks. Bodyweight was measured every other day. Two weeks later, mice were euthanized individually and lesions were excised and processed for disease assessment or immunohistochemistry evaluation. Lesions were measured using a caliper. The extent of endometriosis was evaluated by assessing the total volume of all lesions from each mouse. The volume of the lesions was calculated according to the formula: 0.5 × length × width^2^.

### Histological analyses

Immunofluorescence, immunohistochemistry and H&E staining were performed in paraffin-embedded human or mouse tissue sections. Slides were scanned using a confocal fluorescence microscope (LSM800, ZEISS) or Olympus IX71 fluorescence Microscope and the stain signal was quantified by monitoring the average numbers of positively stained cells from four randomly chosen fields.

### Distribution of BSA-NPs and BSA-GOx-NPs in mice

30 μL 10 mg/ml FITC-BSA-NPs, ICG-BSA-NPs, FITC-BSA-GOx-NPs or ICG-BSA-GOx-NPs were i.p. or i.v. injected into endometriosis mice. After 16 h without further statement, the major organs of mice, including heart, liver, spleen, lung, kidney, eutopic endometrium and ectopic lesions, were collected and fluorescent images of these organs were acquired with IVIS. Moreover, 400 μg anti-Ly6G antibody or an IgG2b isotype control antibody was administered intraperitoneally 24 h prior to injection of FITC-BSA-NPs. Subsequent 200 μg antibody injections were intraperitoneal administered accompanied with FITC- BSA-NPs. Animals were euthanized after 16 h to collect the major organs and fluorescent images were acquired.

### Statistical analysis

All data were analyzed by one-way ANOVA with Turkey’s post hoc test or two-tailed t-test. Statistical analysis was performed using GraphPad Prism 8.0 software.

## Results and discussion

### Continuous enrichment of neutrophils to ectopic lesions

**Fig. 2:**
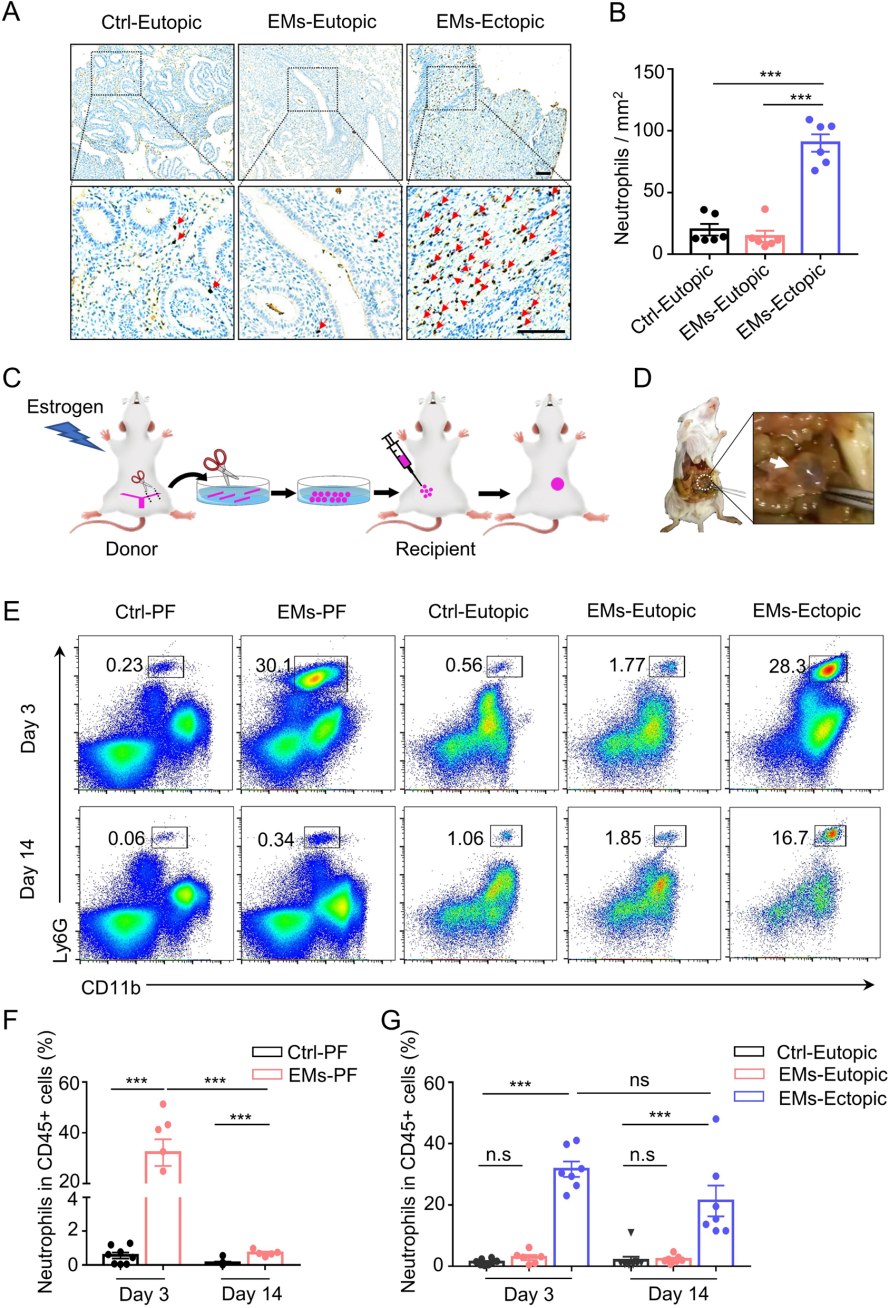
Enrichment of Neutrophils to Ectopic Lesions. **A** Representative MPO immunohistochemistry of ectopic ovarian endometriosis (EMs-Ectopic), matched eutopic endometrium (EMs-Eutopic), and control endometrium (Ctrl-Eutopic). The scale bar is 100 μm. **B** The number of MPO^+^ neutrophils per field in ectopic ovarian endometriosis, matched eutopic endometrium, and control endometrium. *N* = 6 per group. Data are given as the average number of neutrophils in 4 different fields. **C** Schematic illustration of the procedure for the endometriosis model. Estradiol benzoate-treated female mice were sacrificed and uteri were removed and split. Endometrial tissue was isolated and mechanically disrupted before intraperitoneal injection into recipient mice. **D** Images showing fluid-filled endometriotic lesions (circle and arrow) at day 14 after lesion establishment. **E**–**G** Flow cytometric measurements **E** and corresponding quantitative analysis **F** and **G** of the percentage of neutrophils (CD11b^+^Ly6G^+^) in CD45^+^ leukocytes of peritoneal fluid (PF), eutopic endometrium and ectopic lesion at the indicated time points after lesion establishment. *N* = 5 to 9 per time point. Data were shown as mean ± SEM and were analyzed by one-way ANOVA with Turkey’s post hoc test. ***P < 0.001; n.s., not significant

Endometriosis is a chronic inflammatory disease in which neutrophils are increased in the systemic circulation and peritoneal fluid of women with the condition. However, the changes in neutrophils in ectopic lesions with the progression of endometriosis are inconclusive. Therefore, we examined tissue neutrophils from patients with endometriosis by immunohistochemical staining of neutrophil granule myeloperoxidase (MPO). The results showed that neutrophils were significantly increased in ectopic endometriosis compared with either matched eutopic endometrium or control endometrium (Fig. 2A, B). In order to study the changes in neutrophils during the development of endometriosis, we constructed a minimally invasive mouse model of endometriosis by transferring minced endometrial fragments from donor mice into the peritoneum of syngeneic recipient mice as previously described[36, 37] (Fig. 2C). In this model, endometriosis-like lesions can form in diverse peritoneal locations (Fig. 2D), similar to the clinical setting. Using flow cytometric analyses, we found that neutrophils in the peritoneal fluid and ectopic lesions of mice with endometriosis were significantly increased compared to controls at an early stage (day 3) after the establishment of the lesions (Fig. 2E-G). However, no obvious increase in neutrophils was observed in the eutopic endometrium (Fig. 2E, G). Notably, as endometriosis progressed, the frequency of neutrophils in the peritoneal fluid decreased to a level similar to that of controls by day 14 after lesion establishment (Fig. 2F), whereas the frequency of neutrophils in the ectopic lesions remained high (Fig. 2G). These results suggest the presence of a long-term persistent inflammatory microenvironment in endometriotic lesions compared to the peritoneal fluid and ectopic lesions are the major drivers of inflammation in endometriosis, which is consistent with previous research studying the effect of surgical removing endometriotic lesions on the inflammatory profile in women with endometriosis [40]. Given our results demonstrate that neutrophils continue to be enriched in endometriosis lesions as the condition progresses, we speculate that targeting neutrophils may be a promising therapeutic approach for treating endometriosis.

### Highly specific accumulation of intraperitoneally injected BSA-NPs in ectopic lesions

**Fig. 3:**
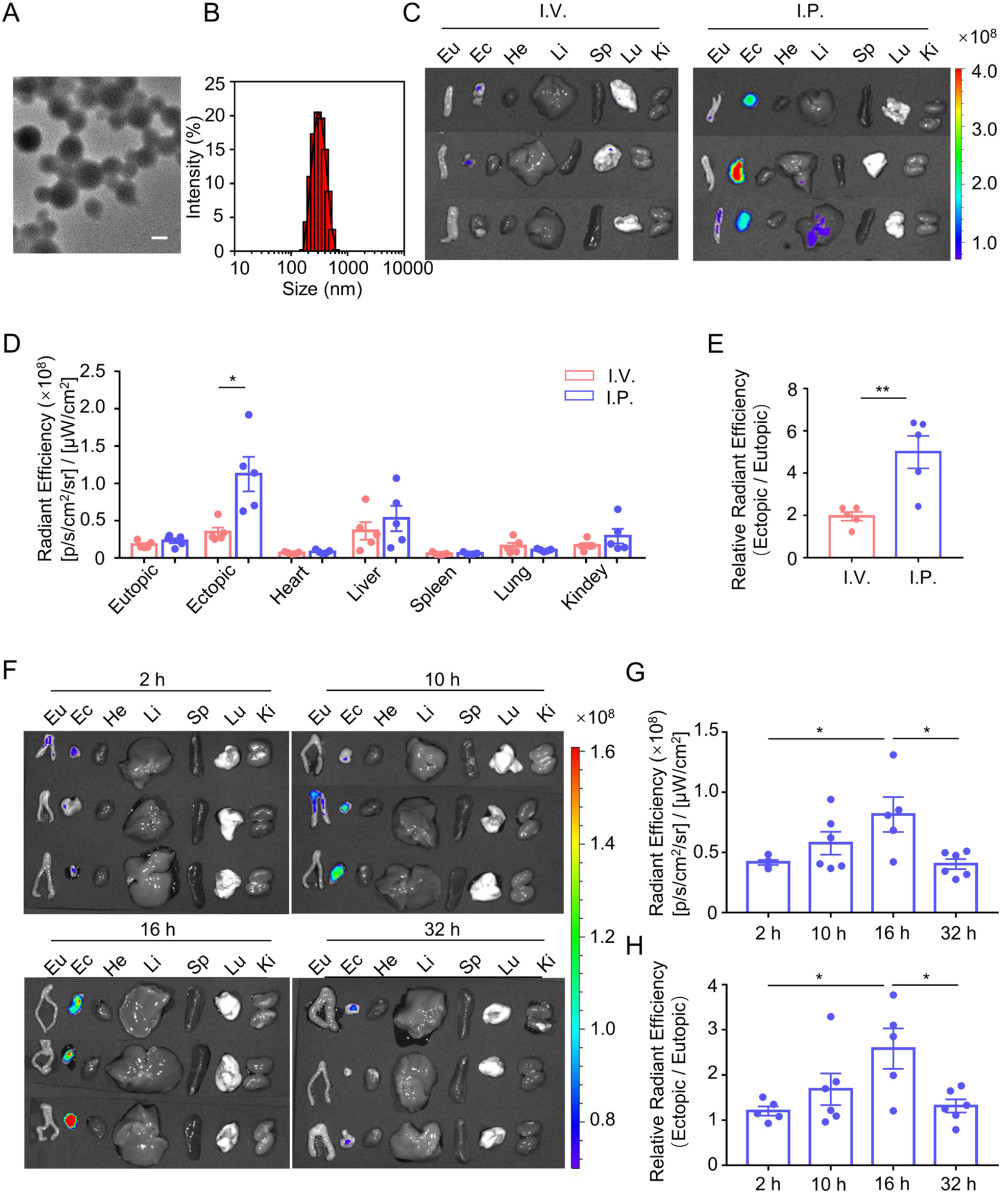
Specific Accumulation of Intraperitoneally Injected BSA-NPs in Ectopic Lesions. **A**, **B**. TEM image **A** and hydrodynamic diameters **B** of BSA-NPs. Scale bar is 200 nm. **C**, **D** Ex vivo fluorescence images **C** and corresponding quantification of average fluorescence intensities of eutopic endometrium, ectopic lesion and major organs **D** collected 16 h post FITC-BSA-NPs injection by intravenous or intraperitoneal, respectively. *N* = 5 per group. **E** Ratio of ectopic/eutopic fluorescence in **D**. *N* = 5 per group. **F**–**H**. Ex vivo fluorescence images **F**, corresponding quantification of average fluorescence intensities of the ectopic lesion **G** and the ratio of ectopic/eutopic fluorescence **H** at different time points post intraperitoneal injection of FITC-BSA-NPs. *N* = 5–6 per group. Data were shown as mean ± SEM and analyzed by one-way ANOVA with Turkey’s post hoc test or two-tailed t-test. *P < 0.05, **P < 0.01

Previous studies investigating the treatment of endometriosis with antibody-mediated neutrophil depletion showed controversial therapeutic effects [8, 9], revealing that neutrophil-targeted therapies require further refinement. Albumin nanoparticles made from denatured BSA were able to hitchhike neutrophils in situ and thus deliver therapeutics to targeted acute inflamed sites [16, 17]. However, the delivery of BSA-NPs to chronically inflamed tissues by neutrophils has not been reported. Therefore, we tested the effects of BSA-NPs in the treatment of endometriosis, a chronic inflammatory disease. We synthesized BSA-NPs using a simple, inexpensive, and reproducible method. Briefly, BSA solution was desolvated with ethanol and then cross-linked with glutaraldehyde to synthesize BSA-NPs. The mean size of the BSA-NPs measured using dynamic light scattering (DLS) and transmission electron microscopy (TEM) was approximately 307 nm (Fig. 3A, B). Subsequently, fluorescein isothiocyanate (FITC)-conjugated BSA-NPs were prepared, and their tissue biodistribution following intravenous or intraperitoneal administration was examined in mice with endometriosis. The highest accumulation of FITC-BSA-NPs in ectopic lesions was observed when BSA-NPs were injected intraperitoneally, whereas FITC-BSA-NPs were rarely detected in ectopic lesions following intravenous injection (Fig. 3C, D). The ratio of fluorescence in ectopic lesions versus eutopic endometrium also reflected a specifically higher distribution in ectopic lesions following intraperitoneal injection (Fig. 3E). We then examined the time-course localization of intraperitoneally injected FITC-BSA-NPs in mice with endometriosis. The results showed that BSA-NPs were consistently enriched to the ectopic lesions for 16 h after injection, and then their enrichment at the lesions decreased at 32 h (Fig. 3F, G). In contrast, no significant changes with increasing time were detected in other organs. The ratio of fluorescence in ectopic lesions versus eutopic endometrium showed similar changes (Fig. 3H).

Considering that FITC is a short-wavelength fluorescent dye and is usually accompanied by high background autofluorescence during in vivo imaging, we chose to label BSA-NPs using a near-infrared (NIR) dye, indocyanine green (ICG), which is an FDA-approved liver function testing dye, to further confirm the biodistribution of BSA-NPs. The results showed that the distribution of ICG-BSA-NPs was similar to that of FITC-BSA-NPs. Significantly more ICG-BSA-NPs fluorescence was detected in ectopic lesions than in eutopic endometrium after intraperitoneal injection (Additional file 1: Fig. S1A). Although intravenous injection of ICG typically leads to high uptake and retention in the liver, intraperitoneal injection significantly reduced the accumulation of ICG-BSA-NPs in the liver compared with intravenous injection (Additional file 1: Fig. S1A, B). Finally, we used the radiant efficiency ratio between different tissues as an index to assess the tissue distribution specificity of ICG-BSA-NPs. The results showed that the ectopic/eutopic ratio increased from 0.95 for intravenous injection to 2.10 for intraperitoneal injection (Additional file 1: Fig. S1C), and the ratio of ectopic/liver increased from 0.11 to 0.93 (Additional file 1: Fig. S1D). These results demonstrate that intraperitoneally injected BSA-NPs are a highly specific delivery platform for endometriotic lesions.

### Neutrophils mediate the accumulation of intraperitoneally injected BSA-NPs in ectopic lesions

**Fig. 4:**
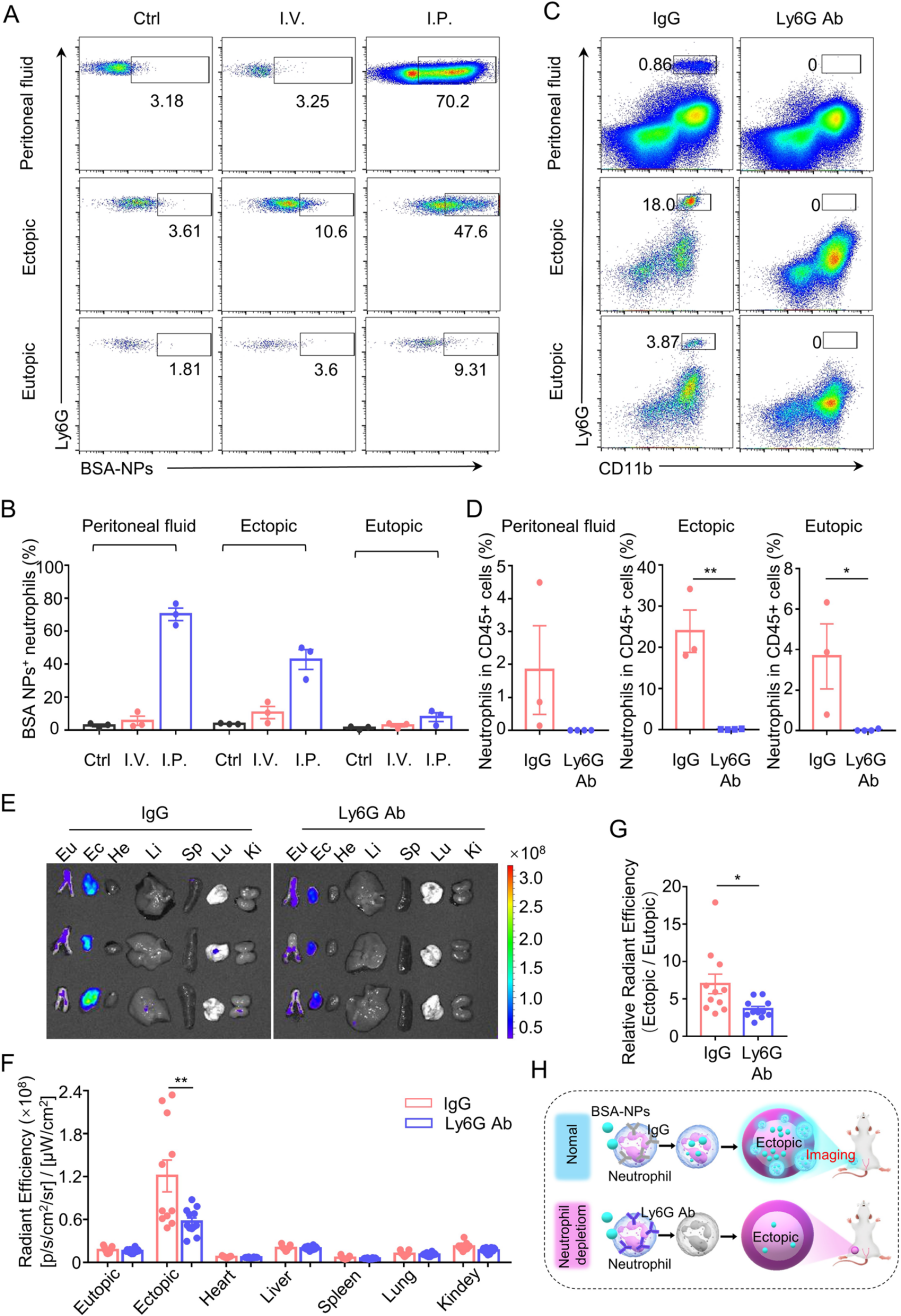
Neutrophil-mediated Accumulation of Intraperitoneally Injected BSA-NPs in Ectopic Lesion. **A**, **B**. Imaging **A** and quantification **B** of FITC-BSA-NPs uptake by neutrophils in peritoneal fluid, ectopic endometrium, and ectopic lesions 16 h after intraperitoneal or intravenous injections. *N* = 3 per group. **C** Representative flow cytometry gating plots showing CD11b^+^ Ly6G^+^ neutrophils in the peritoneal lavage fluid, ectopic endometrium, and ectopic lesions of anti-Ly6G − or isotype control-treated mice. **D** Quantification of CD11b^+^ Ly6G ^+^ neutrophils in the peritoneal lavage fluid, ectopic endometrium, and ectopic lesions of anti-Ly6G − or isotype control-treated mice. *N* = 3 or 4 per group. **E**, **F** Ex vivo fluorescence images **E** and corresponding quantification of average fluorescence intensities of eutopic endometrium, ectopic lesions, and major organs **F** collected from anti-Ly6G − or isotype control-treated mice 16 h post intraperitoneal injection of FITC-BSA-NPs. *N* = 11 per group. **G** The ratio of ectopic/eutopic fluorescence in **F.** *N* = 11 per group. **H** Schematic diagram showing neutrophil-mediated accumulation of BSA-NPs in ectopic lesions. Data were shown as mean ± SEM and analyzed by two-tailed t-test. *P < 0.05, **P < 0.01

To elucidate the mechanism of the highly selective enrichment of intraperitoneally injected BSA-NPs in endometriotic lesions, the role of neutrophils was investigated. We observed that neutrophils take up a large amount of BSA-NP following both intraperitoneal and intravenous injection, but the frequency of BSA-NPs positive neutrophils in the peritoneal fluid after intraperitoneal injection was obviously higher than neutrophils in the blood after intravenous injection (Fig. 4A and Additional file 1: Fig. S2A), which may be attributed to a higher expression of Fcγ receptors in neutrophils in peritoneal fluid than in neutrophils in blood (Additional file 1: Fig. S2B) [16]. Next, we tested the internalization of BSA-NPs in different cells in peritoneal fluid following intraperitoneal injection. We found that, after intraperitoneal injection, the BSA-NPs positive cells were almost CD11b^+^ cells, which include neutrophils and macrophages. Specifically, the frequency of BSA-NPs positive cells in neutrophils, macrophages and CD11b^−^ cells was 63.6%, 46.2% and 1.16%, respectively (Additional file 1: Fig. S3A). Although many BSA-NPs were also internalized into macrophages, there were no significant increase in macrophage recruitment in ectopic lesions compared with eutopic endometrium during the development of endometriosis in mice (Additional file 1: Fig. S3B, C). Moreover, a high frequency of BSA-NPs positive neutrophils was also detected in ectopic lesions (Fig. 4A). These results suggest that neutrophils may play a crucial role in the highly specific delivery of BSA-NPs to ectopic lesions.

To examine this, we specifically depleted neutrophils in vivo using anti-Ly6G antibody. An IgG2b isotype control antibody or anti-Ly6G antibody was administered intraperitoneally 24 h prior to injection of BSA-NPs. Neutrophil depletion was confirmed by flow cytometry analysis of the peritoneal lavage fluid, ectopic endometrial lesions, and eutopic endometrium of the treated mice (Fig. 4C, D). Neutrophil depletion significantly reduced the accumulation of BSA-NPs in ectopic endometrial lesions after intraperitoneal injection (Fig. 4E, F). Moreover, a significant decrease was observed in the ratio of ectopic/eutopic BSA-NPs in neutrophil-depleted mice (Fig. 4G). Taken together, these findings demonstrate that neutrophils mediate the highly specific accumulation of BSA-NPs in ectopic endometrial lesions following intraperitoneal injection (Fig. 4H).

**Fig. 5:**
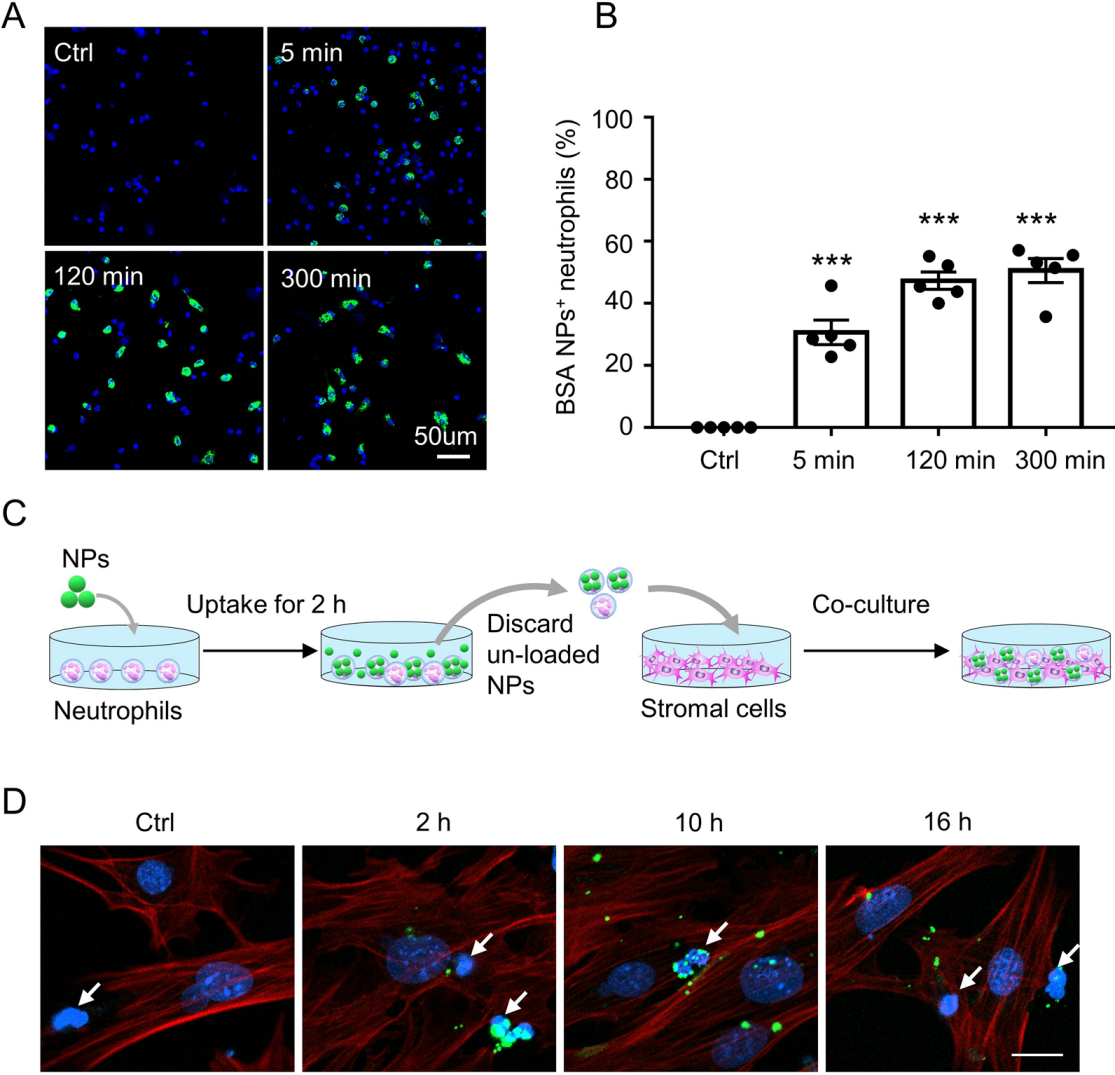
Neutrophils hitchhiking by BSA-NPs. **A** Fluorescent images of neutrophils incubated with BSA-NPs (green) for indicated times. DAPI (blue) was used as a counter staining. Scale is 50 μm. **B** Quantification of FITC-BSA-NPs uptake by neutrophils in **A**. **C** Schematic drawing of the in vitro co-culture system. **D** Fluorescent images of BSA-NPs location after the co-culture of neutrophils and stromal cells for indicated times. Scale is 20 μm. Data are shown as mean ± SEM and were analyzed by one-way ANOVA with Turkey’s post hoc test. ***P < 0.001

Furthermore, the details of neutrophils hitchhiking by BSA-NPs were investigated. We pre-loaded neutrophils with BSA-NPs for indicated times and found that up to 30.65% of neutrophils quickly internalized the BSA-NPs after their co-incubation for 5 min, and the proportion reached 47.31% and 50.59% at 2 h and 5 h, respectively (Fig. 5A, B). Next, we pretreated neutrophils with BSA-NPs for 2 h, and then co-cultured the pretreated neutrophils with primary stromal cells for indicated times to detect the localization of BSA-NPs (Fig. 5C). We observed that most NPs remained resident in neutrophils after 2 h of co-culture, many NPs had been released from neutrophils into stromal cells by 10 h, and almost all NPs were released into stromal cells by 16 h (Fig. 5D).

### Preparation and characterization of BSA-GOx-NPs

**Fig. 6:**
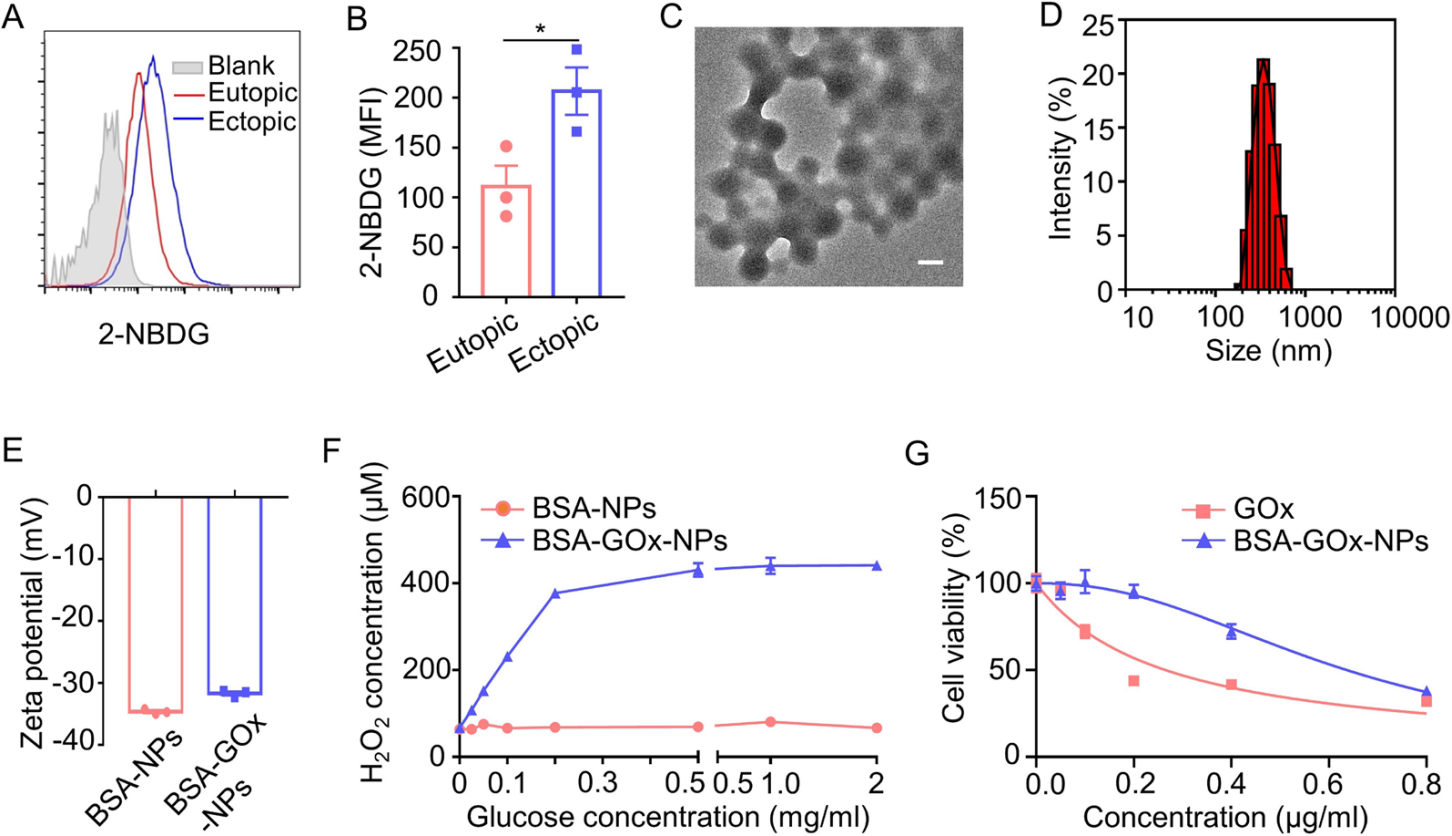
Characterization of BSA-GOx-NPs. **A**, **B** Flow cytometry analysis of the uptake of 2-NBDG by eutopic stromal cells and ectopic stromal cells. All representative histograms **A** were quantified and mean fluorescence values ± SEM **B** are from three independent experiments. Eutopic stromal cells and ectopic stromal cells in each experiment were isolated and pooled from 3 to 4 mice. **C**, **D**. TEM image **C** and hydrodynamic diameters **D** of BSA-GOx-NPs. Scale bar is 200 nm.* N* = 3. **E** Zeta potential of BSA-NPs and BSA-GOx-NPs. *N* = 3 per group. **F** Glucose content-dependent H_2_O_2_ concentrations in “BSA-NPs + glucose” and “BSA-GOx-NPs + glucose” samples. The incubation time for the mixture of BSA-NPs or BSA-GOx-NPs and glucose was 1 h. *N* = 3 per group. **G** Cell viability curves for BSA-GOx-NPs and free GOx.* N* = 3. Data are shown as mean ± SEM and analyzed by two-tailed t-test. *P < 0.05

The previous results suggest that the neutrophil-based BSA-NPs platform may provide an excellent means of targeting an appropriate therapeutic agent for endometriotic lesions. Currently, hormonal therapy is the most common clinical treatment for endometriosis but it is always accompanied by undesirable side effects. In view of this, an alternate therapeutic mechanism was sought. Glucose starvation therapy was considered for our study because of its low side effects. Glucose is the main source of energy in organisms, and we observed increased uptake of the fluorescent glucose analog 2-[N-(7-nitrobenz-2-oxa-1,3-diazol-4-yl) amino]-2-deoxy-D-glucose (2-NBDG) by ectopic stromal cells compared to eutopic stromal cells, suggesting a higher demand for glucose in the ectopic stromal cells (Fig. 6A, B). Glucose oxidase (GOx) is a glucose-consuming enzyme that catalyzes the oxidization of glucose into gluconic acid and hydrogen peroxide (H_2_O_2_), thereby depleting glucose [41]. However, GOx can be easily degraded and deactivated by proteinase during in vivo delivery, which limits its biomedical applications [42, 43]. Interestingly, BSA-NPs are reported to maintain the activity of enzymes such as catalase (CAT) [38]. Based on these findings, we cross-linked BSA and GOx molecules with glutaraldehyde and prepared BSA-GOx-NPs. The statistical results of BSA-Gox-NPs characterization are summarized in Additional file 1: Table S1. BSA-GOx-NPs exhibited a particle size of 334 nm (Fig. 6C, D and Additional file 1: Table S1), and also had a negative charge (Fig. 6E and Additional file 1: Table S1), similar to that of BSA-NPs. Additionally, the polydispersity index (PDI) of BSA-NPs and BSA-GOx-NPs were tested to be 0.039 ± 0.007 and 0.066 ± 0.017 (Additional file 1: Table S1), indicating the good dispersion of nanoparticles. Furthermore, the encapsulation efficiency (EE) and drug loading efficiency (DL) of BSA-GOx-NPs were calculated to be 87.72 ± 2.23% and 8.06 ± 0.20%, respectively (Additional file 1: Table S1 and Fig. S4). To determine the catalytic activity of GOx in the BSA-GOx-NPs, we measured the H_2_O_2_ production catalyzed by BSA-GOx-NPs from glucose. The ammonium titanyl oxalate monohydrate was applied as the indicator of H_2_O_2_. We observed that the H_2_O_2_ generation increased with increasing substrate glucose concentrations (Fig. 6F), indicating the good glucose catalytic capacity of BSA-GOx-NPs. We also tested cell viability following free GOx and BSA-GOx-NPs treatment. The results showed that free GOx exhibited significant cytotoxicity at doses higher than 0.1 μg/ ml, while BSA-GOx-NPs produced cytotoxicity at a dose of 0.4 μg/ ml (Fig. 6G). The calculated IC50s for free GOx and BSA-GOx-NPs were 0.265 and 0.637 μg/ ml, respectively.

### Anti-endometriosis performance of BSA-GOx-NPs

**Fig. 7:**
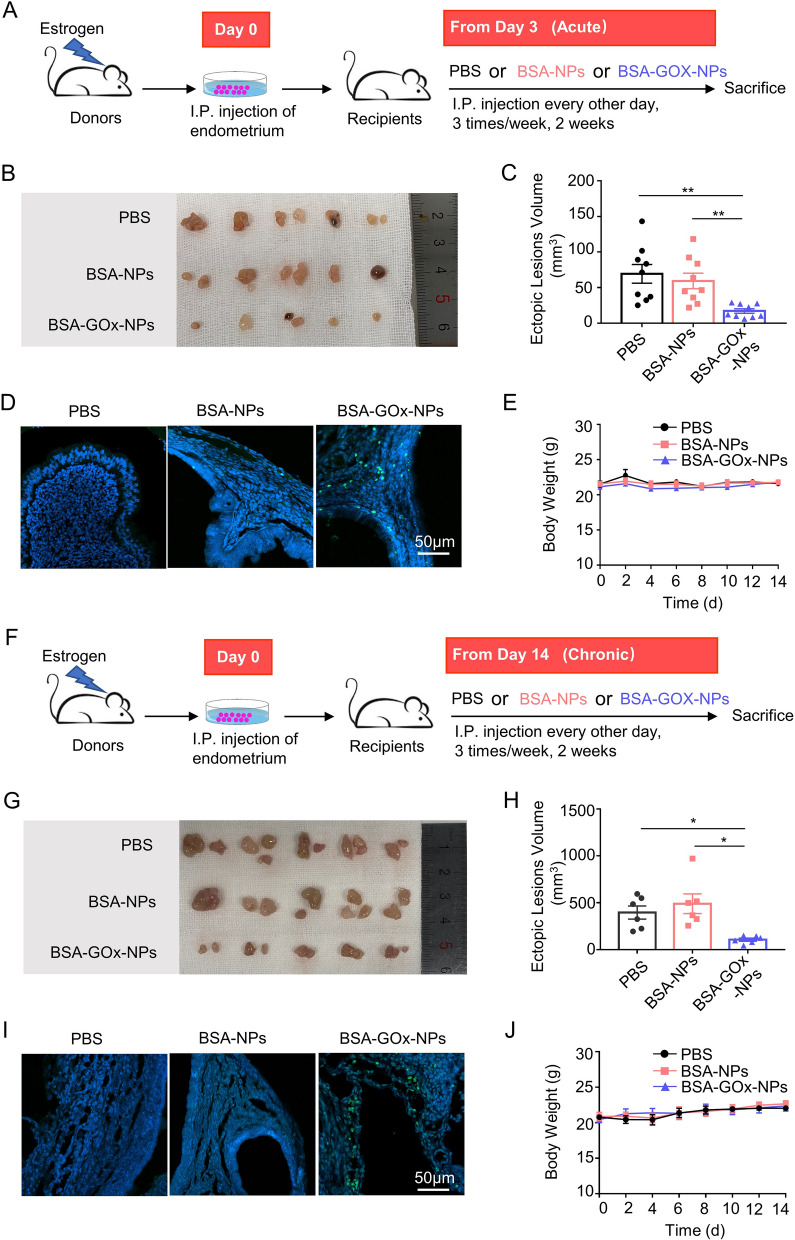
Anti-endometriosis performance of BSA-GOx-NPs. **A** Schematic drawing of treatment with PBS, BSA-NPs or BSA-GOx-NPs, starting from the acute inflammatory phase of endometriosis. **B**, **C**. Images **B** and total volume **C** of endometriotic lesions in mice following different treatments starting from the acute inflammatory phase of endometriosis. *N* = 9 per group. **D** Fluorescence images of TUNEL staining in endometriotic lesions in mice following different treatments starting from the acute inflammatory phase of endometriosis. Blue staining is DAPI, green staining is TUNEL. Scale bar is 50 μm. **E** Monitoring the body weight of mice receiving different treatments starting from the acute inflammatory phase of endometriosis. **F** Schematic drawing of treatment with PBS, BSA-NPs or BSA-GOx-NPs, starting from the chronic inflammatory phase of endometriosis. **G**, **H** Images **G** and total volume **H** of endometriotic lesions in mice following different treatments starting from the chronic inflammatory phase of endometriosis. *N* = 6 per group. **I** Fluorescence images of TUNEL staining in endometriotic lesions in mice following different treatments starting from the chronic inflammatory phase of endometriosis. Blue staining is DAPI, green staining is TUNEL. Scale bar is 50 μm. **J** Monitoring the body weight of mice receiving different treatments starting from the chronic inflammatory phase of endometriosis. Data are shown as mean ± SEM and analyzed by one-way ANOVA with Turkey’s post hoc test or two-tailed t-test. *P < 0.05, **P < 0.01

The efficacy of BSA-GOx-NPs in the treatment of endometriosis was further investigated. The tissue biodistribution of FITC- BSA-GOx-NPs was similar to that of FITC-BSA-NPs, with the highest accumulation in ectopic lesions after intraperitoneal injection, whereas they were rarely detected in ectopic lesions after intravenous injection (Additional file 1: Fig. S5A). Besides, intraperitoneal injection also significantly increased the distribution of ICG-BSA-GOX-NPs in ectopic lesions and decreased their accumulation in the liver, compared with intravenous injection (Additional file 1: Fig. S5B). Next, we randomly divided recipient mice, which were injected with donor mouse endometrial fragments, into three groups, and evaluated the treatment efficacy of BSA-GOx-NPs during the acute (Fig. 7A–E) and chronic (Fig. 7F–J) inflammatory phase of endometriosis, respectively. We found that intraperitoneal injections of BSA-GOx-NPs resulted in smaller lesions, whether treatment was started from the acute or the chronic inflammatory phase (Fig. 7B, G). Statistical analysis showed that the lesion volumes in BSA-GOx-NP-treated mice were significantly smaller than those in PBS and BSA-NPs-treated mice (Fig. 7C, H). Notably, free GOx treatment did not significantly change the volume of ectopic lesions (Additional file 1: Fig. S6). Examination of apoptosis with the terminal deoxynucleotidyl transferase dUTP nick end labeling (TUNEL) assay demonstrated increased green fluorescence signals in BSA-GOx-NPs-treated mice, indicating a higher level of apoptotic cells (Fig. 7D, I). In the studies, no obvious weight loss or pathological changes in major organs were observed in the treated mice after 14 days of dosing (Fig. 7E, J and Additional file 1: Fig. S7). Taken together, the intraperitoneal injection of BSA-GOx-NPs produced an excellent anti-endometriosis effect by GOx-mediated in situ depletion of glucose.

Next, we tested the effect of BSA-GOx-NPs treatment on the populations of neutrophils, stromal cells, macrophages and T cells in ectopic lesions in vivo. No significant differences of neutrophils were detected under different treatments (Additional file 1: Fig. S8), suggesting that the anti-endometriosis effect of BSA-GOx-NPs might not be exerted by reducing the enrichment of neutrophils to the lesions. Besides, the population of macrophages and T cells in the mice lesions did not show significant differences under different treatments (Additional file 1: Fig. S8), further excluding the anti-endometriosis efficacy of BSA-GOx-NPs by mobilizing the immune system. Moreover, the stroma cells population was remarkably reduced in the lesions of BSA-GOx-NPs-treated mice compared with those under PBS- or BSA-NPs-treated mice (Additional file 1: Fig. S8). Combined with the apparent apoptosis in the lesions of BSA-GOx-NPs-treated mice (Fig. 7D, I), we believe that BSA-GOx-NPs were more likely to directly induce apoptosis in ectopic lesions rather than indirectly kill ectopic cells by mobilizing the immune system.

## Conclusion

In summary, neutrophils were consistently enriched in ectopic lesions in a mouse model and patients with endometriosis. Based on these findings, we synthesized a neutrophil hitchhiking BSA-NPs and found that intraperitoneal injection of BSA-NPs led to high specific enrichment in ectopic lesions in a neutrophil-dependent manner. Furthermore, more glucose uptake was detected in ectopic stromal cells compared to eutopic stromal cells. Thus, glucose oxidase (GOx) was loaded into BSA-NPs and intraperitoneally injection of BSA-GOx-NPs demonstrated excellent anti-endometriosis effects with no observable side effects. These results reveal for the first time that the neutrophil hitchhiking strategy is effective in a model of chronic inflammatory disease and provide a non-hormonal, easy-to-achieve, and repeatable approach for the treatment of endometriosis.

## Supplementary Information

**Additional file 1: Table S1.** Characteristics of Blank BSA-NPs and BSA-GOx-NPs. **Figure S1.** The biodistribution of ICG -BSA-NPs *in vivo*. A, B). Ex vivo fluorescence images (A) and corresponding quantification of average fluorescence intensities of eutopic endometrium, ectopic lesions and major organs (B) collected 16 h post ICG-BSA-NPs intravenous or intraperitoneal injection, respectively. C, D). Ratio of ectopic/eutopic (C) and ectopic/liver (D) fluorescence in (B). N =7 per group. Data are shown as mean ± SEM and were analyzed by analyzed by two-tailed t test. *P < 0.05, **P < 0.01, ***P < 0.001. **Figure S2.** A). Representative flow cytometry imaging of FITC-BSA-NPs uptake by neutrophils in blood 30 min after intraperitoneal or intravenous injections. B).FcγR III expression on the surface of neutrophils in blood and in peritoneal fluid. **Figure S3**. A). Internalization of FITC-BSA-NPs by CD11b^-^ cells, neutrophils and macrophages in peritoneal fluid after the administration of FITC-BSA-NPs. B-C). Flow cytometric measurements (B) and corresponding quantitative analysis (C) of percentage of macrophages (CD11b^+^F4/80^+^) in CD45^+^ leukocytes of peritoneal fluid (PF), eutopic endometrium and ectopic lesion at the indicated time points after lesion establishment. *N* = 4 per time point. Data were shown as mean ± SEM and were analyzed by one-way ANOVA with Turkey’s post hoc test. *P < 0.05. **Figure S4.** Linear fitting of the fluorescence intensity of GOx-FITC (excitation at 488 nm, emission at 514 nm) versus the GOx concentration. Figure S5. The biodistribution of BSA-GOx-NPs *in vivo*. Ex vivo fluorescence images of eutopic endometrium, ectopic lesion and major organs collected 16 h post FITC-BSA-GOx-NPs injection (A) or ICG-BSA-GOx-NPs (B) by intraperitoneal or intravenous respectively. **Figure S6.** A, B). Images (A) and total volume (B) of endometriotic lesions in mice following different treatments starting from the acute inflammatory phase of endometriosis. *N*= 5 per group. C). Monitoring the body weight of mice receiving different treatments starting from the acute inflammatory phase of endometriosis. Data are shown as mean ± SEM and analyzed by one-way ANOVA with Turkey’s post hoc test or two-tailed t test. *P < 0.05. **Figure S7.** HE staining images of major organs. Mice with endometriosis were treated with sterile PBS, BSA -NPs and BSA-GOx-NPs. Two weeks later, mice were sacrificed and the main organs were taken for HE staining. Scale bar is 100 μm. **Figure S8.** The effect of BSA-GOx-NPs treatment on the populations of neutrophils, stromal cells, macrophages and T cells in ectopic lesions *in vivo.* Mice with endometriosis were treated with sterile PBS, BSA -NPs and BSA-GOx-NPs. Two weeks later, mice were sacrificed and the ectopic lesions were taken for staining. Scale bar is 50 μm. Data are shown as mean ± SEM and were analyzed by one-way ANOVA with Turkey’s post hoc test. **P < 0.01, ***P < 0.001.

## Data Availability

All data generated and analyzed during this research are included in this published article and its additional file.
