# Supplementary material for: Highly specific neutrophil-mediated delivery of albumin nanoparticles to ectopic lesion for endometriosis therapy
Source: J Nanobiotechnology. 2023 Mar 8;21:81. doi: 10.1186/s12951-023-01831-4 (PMC9996962; doi:10.1186/s12951-023-01831-4)
Supplement: Supplementary file 1 — Additional file 1: Table S1. Characteristics of Blank BSA-NPs and BSA-GOx-NPs. Figure S1. The biodistribution of ICG -BSA-NPs in vivo. A, B). Ex vivo fluorescence images (A) and corresponding quantification of average fluorescence intensities of eutopic endometrium, ectopic lesions and major organs (B) collected 16 h post ICG-BSA-NPs intravenous or intraperitoneal injection, respectively. C, D). Ratio of ectopic/eutopic (C) and ectopic/liver (D) fluorescence in (B). N =7 per group. Data are shown as mean ± SEM and were analyzed by analyzed by two-tailed t test. *P < 0.05, **P < 0.01, ***P < 0.001. Figure S2. A). Representative flow cytometry imaging of FITC-BSA-NPs uptake by neutrophils in blood 30 min after intraperitoneal or intravenous injections. B).FcγR III expression on the surface of neutrophils in blood and in peritoneal fluid. Figure S3. A). Internalization of FITC-BSA-NPs by CD11b- cells, neutrophils and macrophages in peritoneal fluid after the administration of FITC-BSA-NPs. B-C). Flow cytometric measurements (B) and corresponding quantitative analysis (C) of percentage of macrophages (CD11b+F4/80+) in CD45+ leukocytes of peritoneal fluid (PF), eutopic endometrium and ectopic lesion at the indicated time points after lesion establishment. N = 4 per time point. Data were shown as mean ± SEM and were analyzed by one-way ANOVA with Turkey’s post hoc test. *P < 0.05. Figure S4. Linear fitting of the fluorescence intensity of GOx-FITC (excitation at 488 nm, emission at 514 nm) versus the GOx concentration. Figure S5. The biodistribution of BSA-GOx-NPs in vivo. Ex vivo fluorescence images of eutopic endometrium, ectopic lesion and major organs collected 16 h post FITC-BSA-GOx-NPs injection (A) or ICG-BSA-GOx-NPs (B) by intraperitoneal or intravenous respectively. Figure S6. A, B). Images (A) and total volume (B) of endometriotic lesions in mice following different treatments starting from the acute inflammatory phase of endometriosis. N= 5 per group. C). Monit [file 12951_2023_1831_MOESM1_ESM.docx]

**Additional Materials**

**Table S1.** Characteristics of Blank BSA-NPs and BSA-GOx-NPs


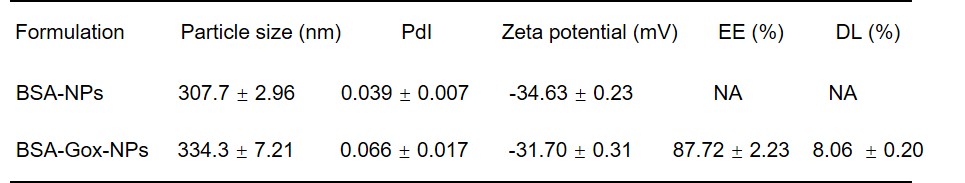


**Additional Figure 1**


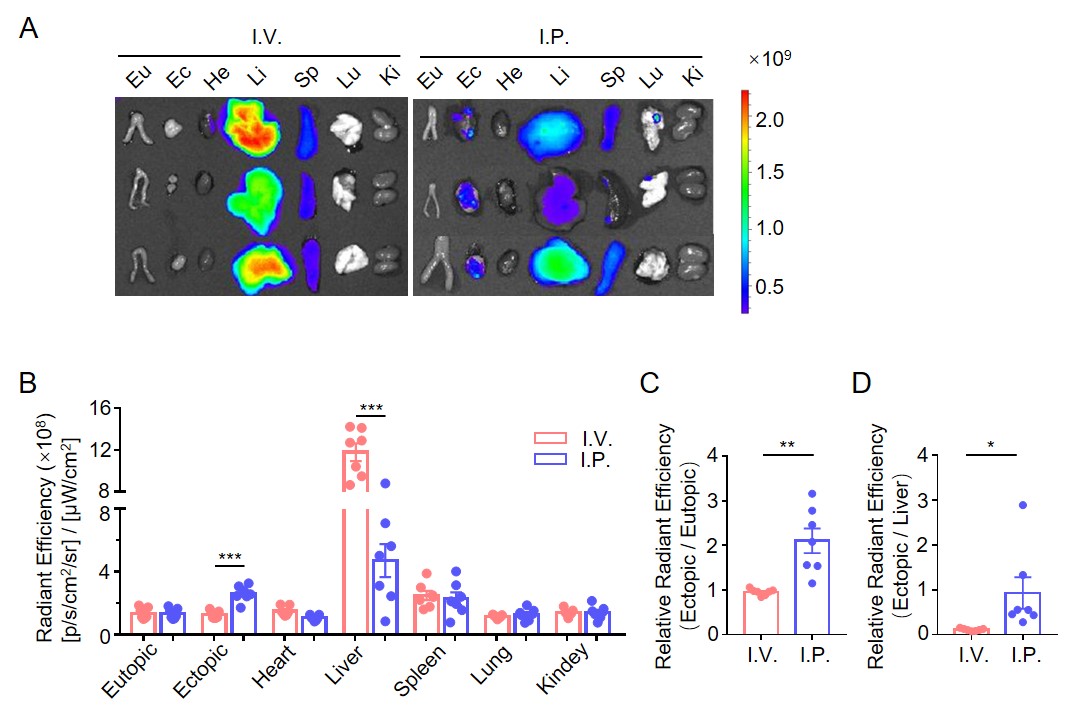


**The biodistribution of ICG -BSA-NPs *in vivo*.** A, B). Ex vivo fluorescence images (A) and corresponding quantification of average fluorescence intensities of eutopic endometrium, ectopic lesions and major organs (B) collected 16 h post ICG-BSA-NPs intravenous or intraperitoneal injection, respectively. C, D). Ratio of ectopic/eutopic (C) and ectopic/liver (D) fluorescence in (B). N =7 per group. Data are shown as mean ± SEM and were analyzed by analyzed by two-tailed t test. *P < 0.05, **P < 0.01, ***P < 0.001.

**Additional Figure 2**


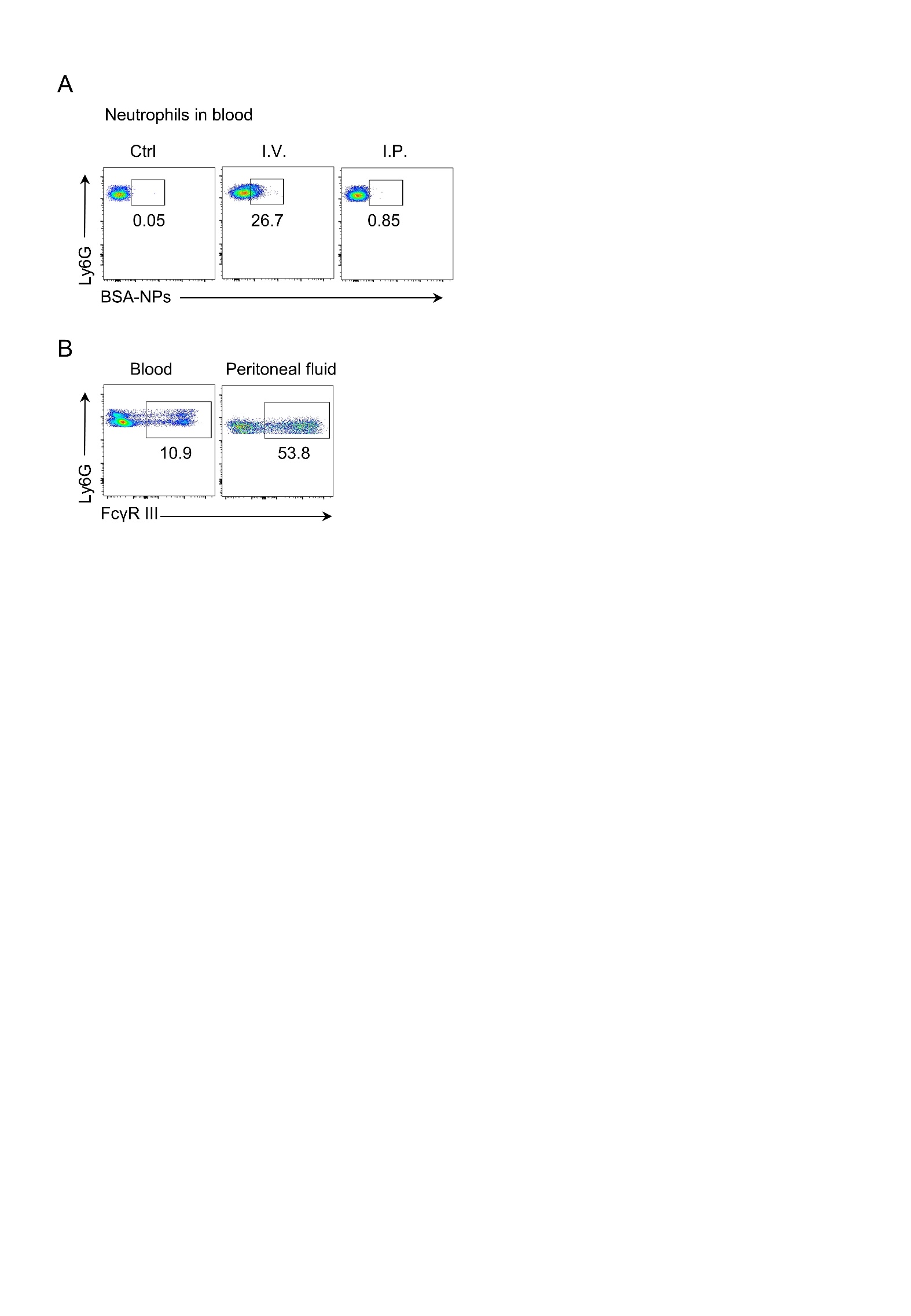


A). Representative flow cytometry imaging of FITC-BSA-NPs uptake by neutrophils in blood 30 min after intraperitoneal or intravenous injections. B).FcγR III expression on the surface of neutrophils in blood and in peritoneal fluid.

**Additional Figure 3**


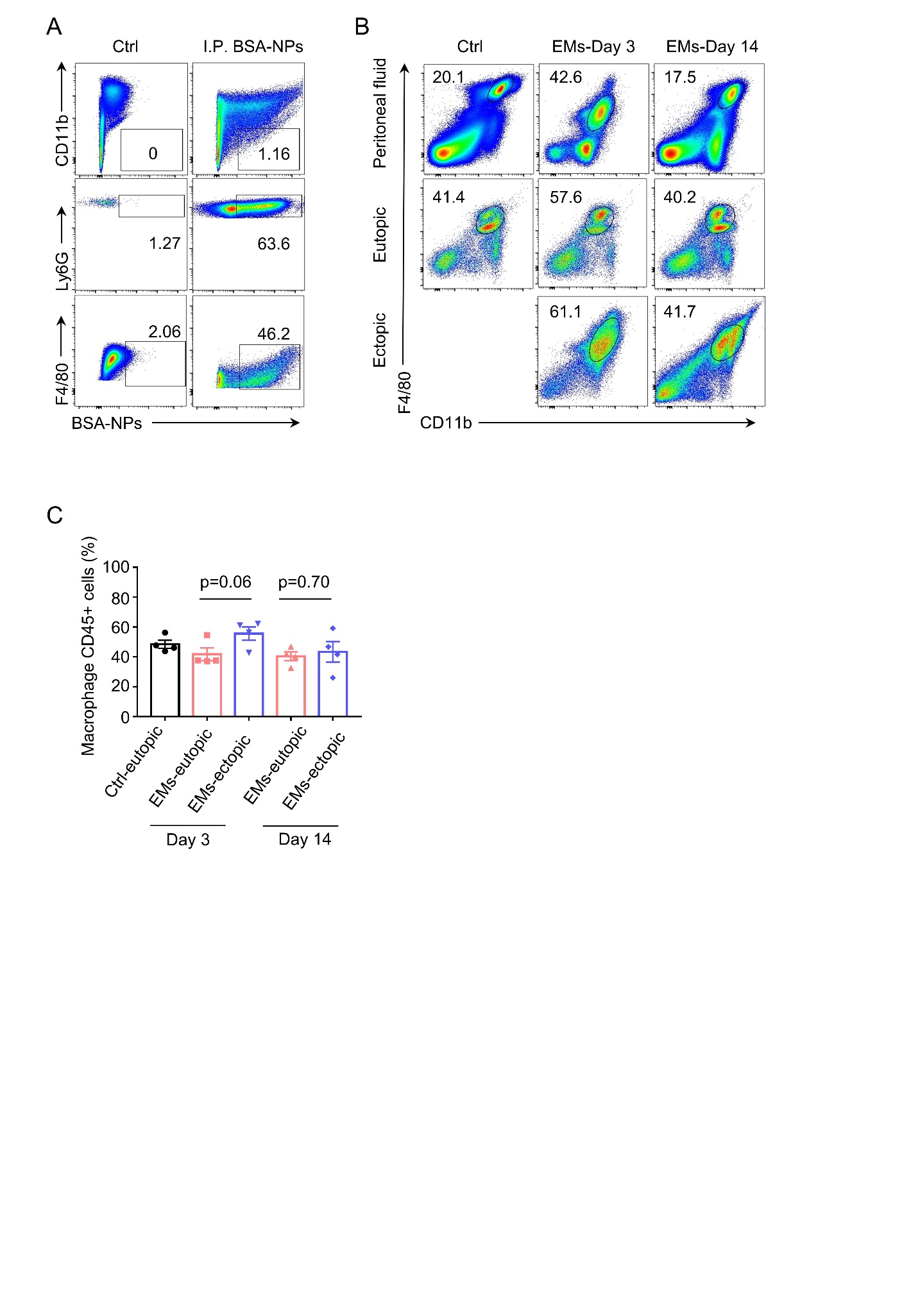


A). Internalization of FITC-BSA-NPs by CD11b^-^ cells, neutrophils and macrophages in peritoneal fluid after the administration of FITC-BSA-NPs. B-C). Flow cytometric measurements (B) and corresponding quantitative analysis (C) of percentage of macrophages (CD11b^+^F4/80^+^) in CD45^+^ leukocytes of peritoneal fluid (PF), eutopic endometrium and ectopic lesion at the indicated time points after lesion establishment. *N* = 4 per time point. Data were shown as mean ± SEM and were analyzed by one-way ANOVA with Turkey’s post hoc test. **P* < 0.05.

**Additional Figure 4**


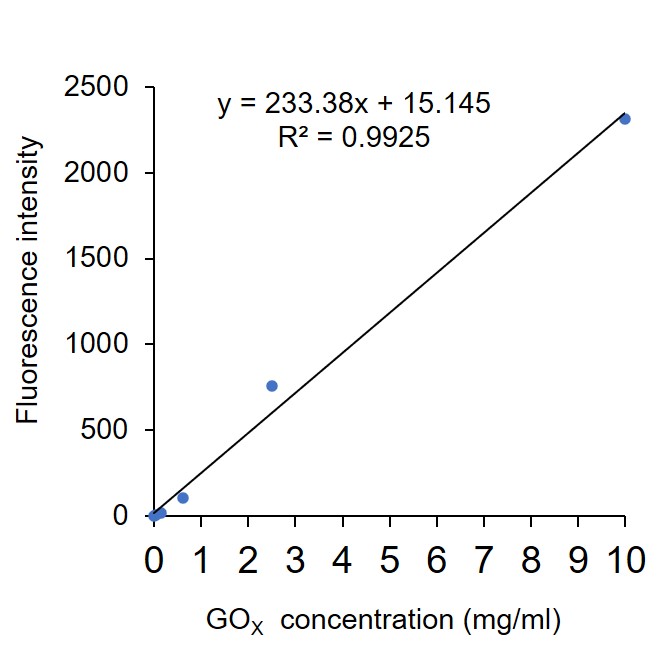


Linear fitting of the fluorescence intensity of GOx-FITC (excitation at 488 nm, emission at 514 nm) versus the GOx concentration.

**Additional Figure 5**


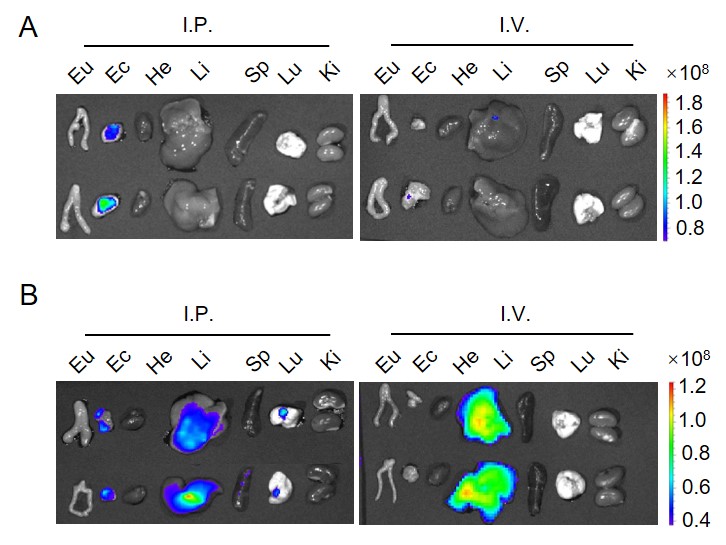


**The biodistribution of BSA-GOx-NPs *in vivo*.** Ex vivo fluorescence images of eutopic endometrium, ectopic lesion and major organs collected 16 h post FITC-BSA-GOx-NPs injection (A) or ICG-BSA-GOx-NPs (B) by intraperitoneal or intravenous respectively.

**Additional Figure 6**


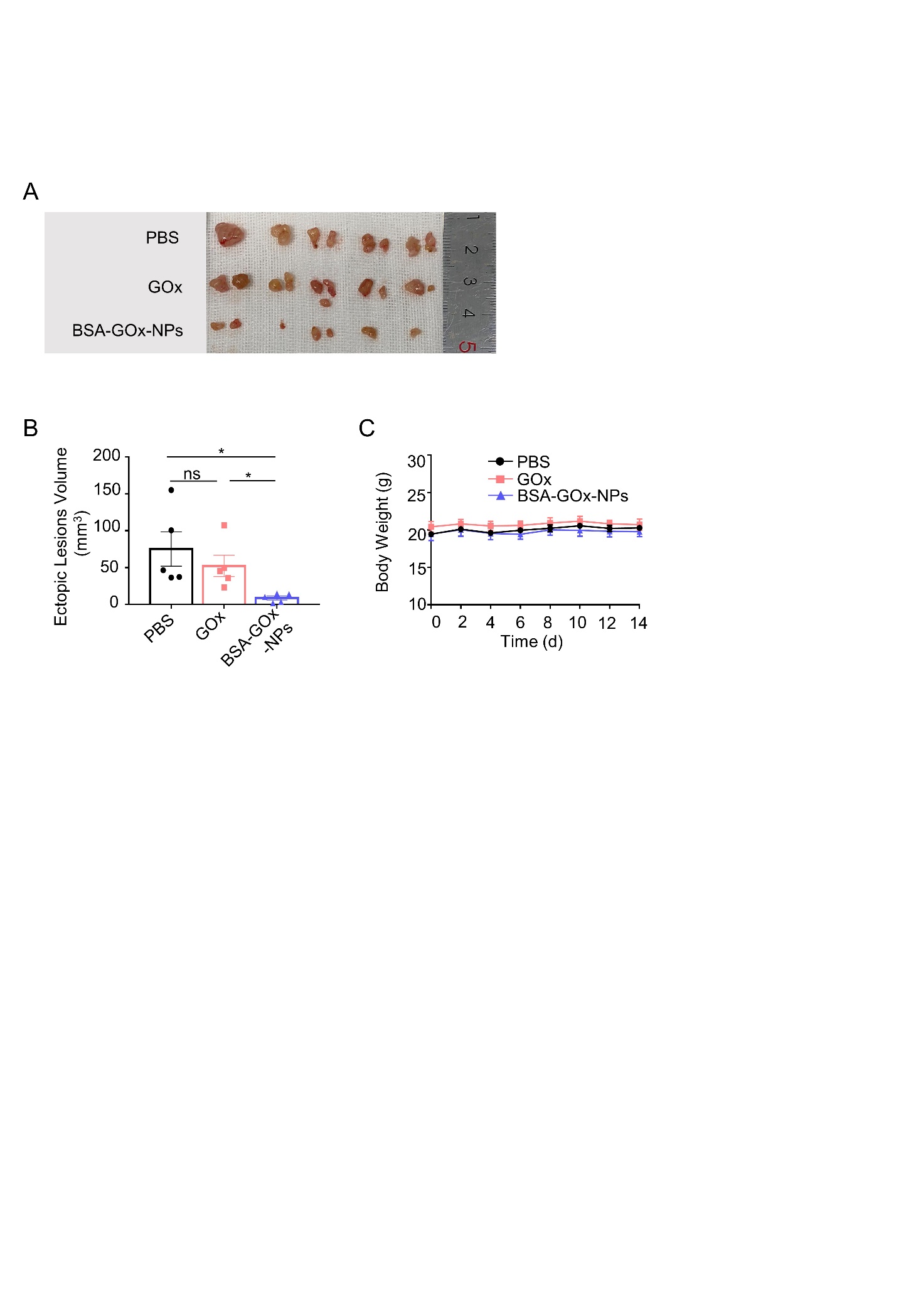


A, B). Images (A) and total volume (B) of endometriotic lesions in mice following different treatments starting from the acute inflammatory phase of endometriosis. *N*= 5 per group. C). Monitoring the body weight of mice receiving different treatments starting from the acute inflammatory phase of endometriosis. Data are shown as mean ± SEM and analyzed by one-way ANOVA with Turkey’s post hoc test or two-tailed t test. **P* < 0.05.

**Additional Figure 7**


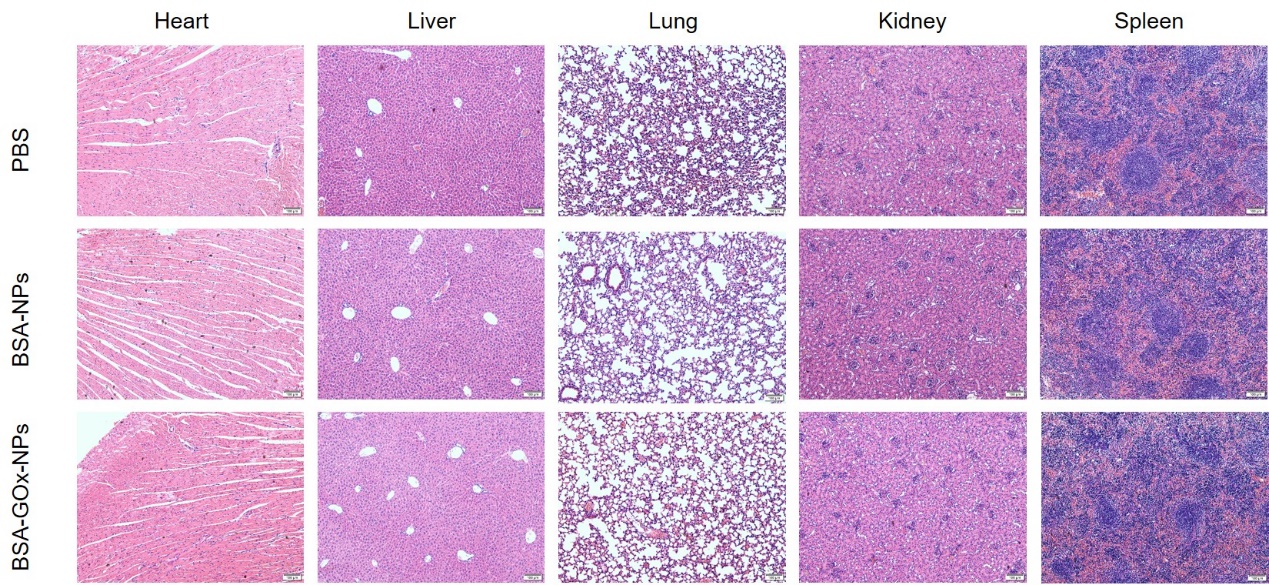


**HE staining images of major organs.** Mice with endometriosis were treated with sterile PBS, BSA -NPs and BSA-GOx-NPs. Two weeks later, mice were sacrificed and the main organs were taken for HE staining. Scale bar is 100 μm.

**Additional Figure 8**


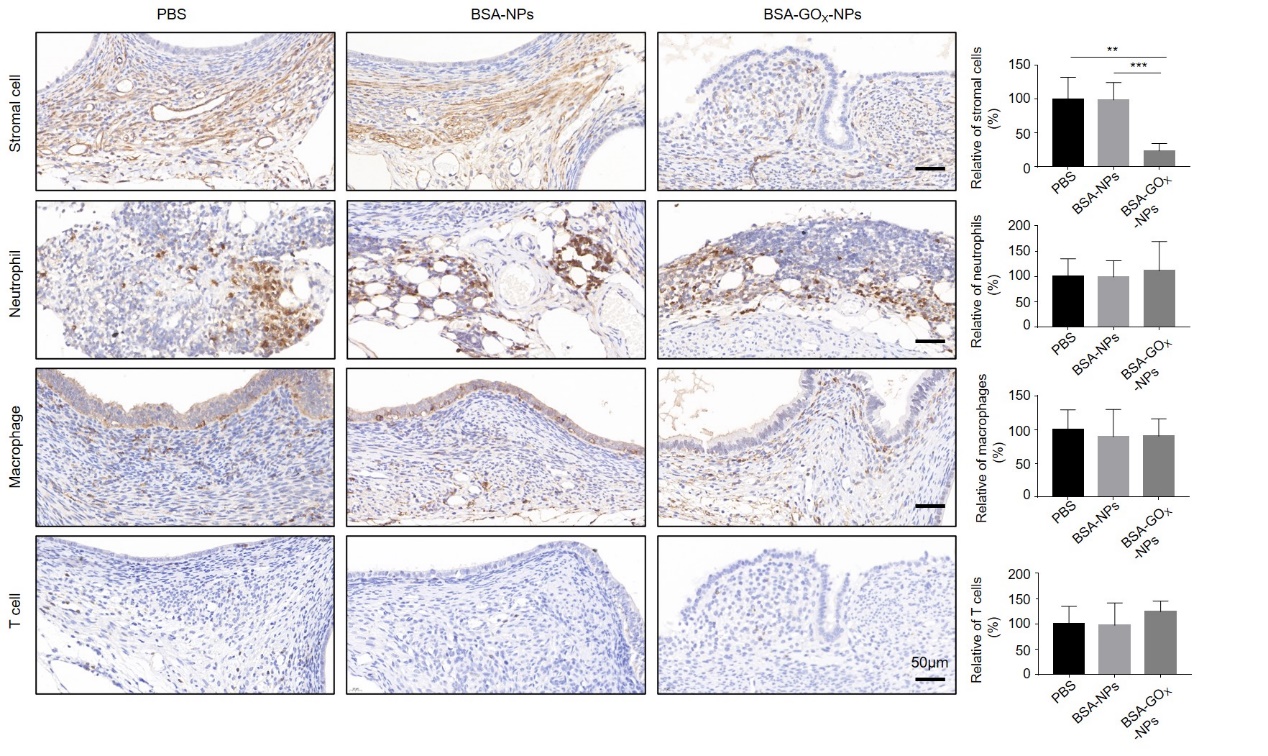


**The effect of BSA-GOx-NPs treatment on the populations of neutrophils, stromal cells, macrophages and T cells in ectopic lesions *in vivo.*** Mice with endometriosis were treated with sterile PBS, BSA -NPs and BSA-GOx-NPs. Two weeks later, mice were sacrificed and the ectopic lesions were taken for staining. Scale bar is 50 μm. Data are shown as mean ± SEM and were analyzed by one-way ANOVA with Turkey’s post hoc test. **P < 0.01, ***P < 0.001.
